# Characterization of 150 Wheat Cultivars by LC-MS-Based Label-Free Quantitative Proteomics Unravels Possibilities to Design Wheat Better for Baking Quality and Human Health

**DOI:** 10.3390/plants10030424

**Published:** 2021-02-24

**Authors:** Muhammad Afzal, Malte Sielaff, Valentina Curella, Manjusha Neerukonda, Khaoula El Hassouni, Detlef Schuppan, Stefan Tenzer, C. Friedrich H. Longin

**Affiliations:** 1 State Plant Breeding Institute, University of Hohenheim, Fruwirthstr. 21, 70599 Stuttgart, Germany; m.afzal@uni-hohenheim.de (M.A.); khaoula.elhassouni@uni-hohenheim.de (K.E.H.); 2 Institute for Immunology, University Medical Center of the Johannes Gutenberg University Mainz, Langenbeckstr. 1, 55131 Mainz, Germany; malte.sielaff@uni-mainz.de (M.S.); tenzer@uni-mainz.de (S.T.); 3 Institute of Translational Immunology, University Medical Center of the Johannes Gutenberg University Mainz, Langenbeckstr. 1, 55131 Mainz, Germany; cvalenti@uni-mainz.de (V.C.); manjusha@uni-mainz.de (M.N.); detlef.schuppan@unimedizin-mainz.de (D.S.); 4 Division of Gastroenterology, Beth Israel Deaconess Medical Center, Harvard Medical School, 330 Brookline Avenue, Boston, MA 02215, USA

**Keywords:** LC-MS proteomics, wheat, healthy nutrition, future breeding

## Abstract

Wheat (*Triticum aestivum* ssp. *aestivum*) contributes to 20% of the human protein supply, delivers essential amino acids and is of fundamental importance for bread and pasta quality. Wheat proteins are also involved in adverse human reactions like celiac disease (CD), wheat allergy (WA) and non-celiac wheat sensitivity (NCWS). Using liquid chromatography-mass spectrometry (LC-MS)-based label-free quantitative (LFQ) proteomics of aqueous flour extracts, we determined 756 proteins across 150 wheat cultivars grown in three environments. However, only 303 proteins were stably expressed across all environments in at least one cultivar and only 89 proteins thereof across all 150 cultivars. This underlines the large influence of environmental conditions on the expression of many proteins. Wheat cultivars varied largely in their protein profile, shown by high coefficients of variation across different cultivars. Heritability (h^2^) ranged from 0–1, with 114 proteins having h² > 0.6, including important proteins for baking quality and human health. The expression of these 114 proteins should be amenable to targeted manipulation across the wheat supply chain by varietal choice and breeding for designing healthier wheat with better quality. Further technical development is urgently required to assign functions to identifiable proteins labeled yet uncharacterized in databases and speeding up detection methods to routinely use proteomics in wheat supply chains.

## 1. Introduction

Wheat (*Triticum aestivum* ssp. *aestivum*) is grown globally in over 120 countries, covering 16% of the cultivated land area, and is therefore a key source of carbohydrates and proteins for growing populations (https://www.wheatinitiative.org/, accessed on 3 October 2020). Protein contents in wheat grains depend on the chosen cultivar due to significant genetic variations [1], to contrasting fertilizer management and to environmental factors such as soil, rainfall and temperature, all affecting the nutrients uptake [2,3,4]. Furthermore, the grain protein content is negatively correlated with grain yield and grain starch content [5]. On average, wheat grain has a protein content of 8–13% [6], which is contributed by water/salt-soluble albumins and globulins (15–20%) and gluten proteins (80–85%), the latter serving as storage proteins and endowing wheat products with the desired viscoelastic and gustatory properties [7,8,9].

Importantly, some wheat proteins can cause adverse inflammatory reactions in humans: (1) certain gluten peptide sequences trigger small intestinal and extraintestinal T cell activation and inflammation in patients with celiac disease [10,11]; (2) the family of wheat alpha-amylase/trypsin inhibitors (ATIs) stimulate intestinal innate immune cells via the activation of Toll-like receptor 4 and promote intestinal and extraintestinal inflammation [12,13,14,15,16,17]; (3) numerous wheat albumins and globulins, such as serpins, lipid transfer proteins, β-amylases and ATIs, as well as a few gluten proteins, can elicit respiratory and nutritional immediate allergic reactions [18,19,20,21]; and (4) a novel form of nutritional wheat allergy with an immediate intestinal, but a delayed clinical, reaction to wheat proteins is highly prevalent among patients with “irritable bowel syndrome” [22,23]. The first studies showed that wheat cultivars largely differ in the compositions of ATIs [24,25] or of the 33-mer α-gliadin peptide, a key immunogen for T cells in patients with celiac disease [26]. However, investigations of a large number of proteins across many wheat cultivars grown under different environmental conditions are lacking.

Already, in the 1980s, using gel electrophoresis, the compositions of glutenins and gliadins across a range of wheat cultivars and their effects on bread-making quality were assessed [27,28]. Using mass spectrometry, recently, it was shown that the contents of gliadins, glutenins and albumins/globulins varied across 60 wheat cultivars [29]. Moreover, it has been reported that wheat breeding has modified the gluten composition and that recent wheat cultivars contain higher amounts of high and low molecular weight glutenins (HMW and LMW) than older wheat cultivars [29]. However, to date, these proteomic methods depend on quantitative extraction and do not allow a “high-throughput” quantification of a large spectrum of functionally relevant wheat proteins. 

During the last decade, the technology of mass spectrometry has been revolutionized [30], permitting a detailed analysis of the whole proteomes of eukaryotes [31], including humans [32] and plants [33]. With the publication of the wheat reference sequence [34] and its recent extension to 15 fully sequenced wheat cultivars [35], the wheat proteome can now be characterized at a high resolution. Thus, Afzal et al. [36] identified 3050 and 2770 proteins in 15 cultivars of spelt and wheat, respectively, and showed that the majority of these proteins were mainly affected by the environment. However, about 300 proteins had an intermediate-to-high heritability and large coefficients of variation, indicating that their expression levels could be changed through the varietal choice.

Here, we extended our prior study using liquid chromatography-mass spectrometry (LC-MS)-based label-free quantitative (LFQ) proteomics to quantify the extracts of the functionally relevant wheat albumins and globulins from 150 wheat cultivars registered between 1921 and 2013 that were grown in parallel in three diverse German environments. To evaluate the potential of proteomics for future wheat trading and breeding, we investigated, in particular, (1) the magnitude of environmental versus genetic influence on protein expression, (2) the variance components and the heritability and correlation of these proteins to important wheat quality traits and (3) temporal trends of the detected proteins across wheat breeding history from 1921 to 2013.

## 2. Results

**Figure 1:**
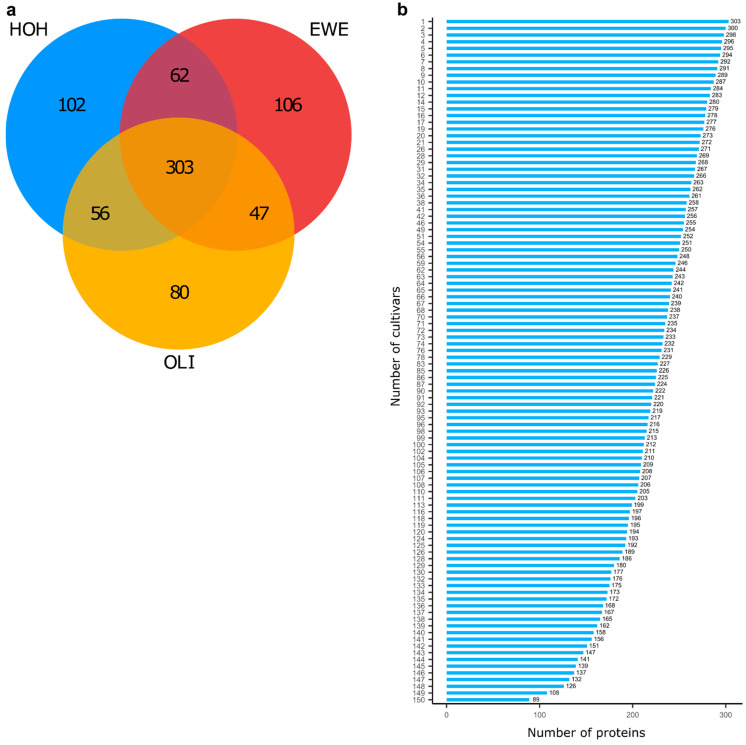
The protein expression is influenced by the environment and cultivar. A number of proteins (756) were identified in 150 cultivars grown under three diverse environments—HOH (Hohenheim), EWE (Eckartsweier) and OLI (Oberer Lindenhof), but only 303 proteins were stably expressed across all three environments in at least one cultivar, while 62, 47 and 56 proteins were expressed in two environments and 102, 106 and 80 proteins only in a single environment (**a**). From the 303 proteins, which were stably expressed in all environments in at least one cultivar, only 89 were stably expressed in all cultivars, while the abundance of 214 differed depending on the chosen cultivar (**b**).

Using LFQ proteomics, we identified 756 proteins from aqueous extracts of wheat flours across 150 cultivars, which were grown in three environments. Notably, the number of detected proteins varied largely across the environments, although the same set of cultivars were grown under similar agricultural practices (Figure 1a). At the single locations of Hohenheim (HOH), Eckartsweier (EWE) and Oberer Lindenhof (OLI), 523, 518 and 486 proteins were expressed, respectively. Moreover, 102, 106 and 80 proteins were only expressed in HOH, EWE and OLI, respectively. On the other hand, 303 proteins were expressed across all environments in at least one cultivar and 89 in all 150 cultivars (Figure 1b). For further analyses, we focused on the 303 proteins that were stably expressed in at least one cultivar across all three environments. 

**Figure 2:**
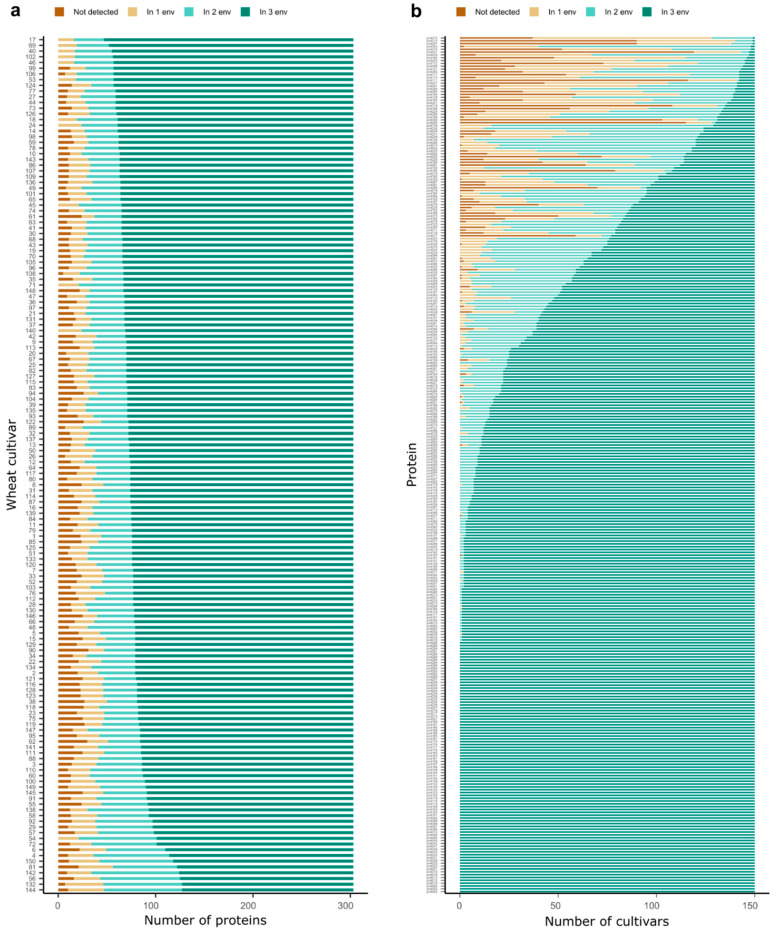
Protein–cultivar–environment interactions. The number of proteins expressed in each single cultivar (**a**) or the number of cultivars with an expression of each single protein (**b**) in none, 1, 2 or 3 environments, respectively. env: environment.

The proteome profiles of the different cultivars varied considerably either in the number of expressed proteins or in terms of the number of environments in which the particular proteins were stably expressed (Figure 2a,b). In a few cultivars, only about 55% of the proteins were stably expressed across three environments, e.g., in the cultivars Akteur (No. 56), Colonia (No. 132) and Florida (No. 144), while, in other cultivars, this value was close to 80%, e.g., in the cultivars Chevalier (No. 17), Lear (No. 40) and Carenius (No. 69). Similarly, some proteins were present consistently across the three environments only in a single or a couple of cultivars, while, in other cultivars, they were expressed only in two, one or even no environments, e.g., prot273 (A0A3B5Z2Q8), prot212 (R4ZC73), prot207 (A0A3B6ESH8) and prot303 (K7WV92). On the other hand, many proteins were detected across the three environments in all cultivars, e.g., prot002 (Q9ZR70), prot003 (W4ZP51), prot005 (A0A3B6IX62) and prot010 (A0A3B6RKV2).

**Figure 3:**
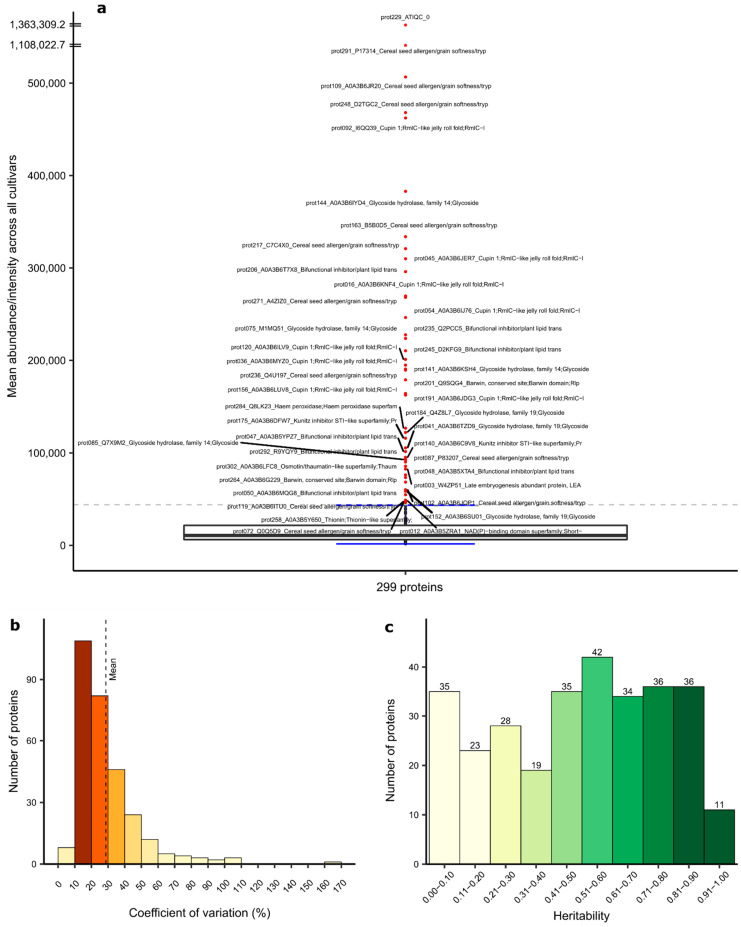
Best Linear Unbiased Estimates (BLUEs)—the annotation labels contain the internal protein number, followed by the UniProt accession and InterPro protein name; red points denote proteins with a relatively high mean abundance (**a**), coefficient of variation (**b**) and heritability (**c**) of the expression level of 299 proteins, which were stably expressed in all the environments in at least one cultivar.

**Figure 4:**
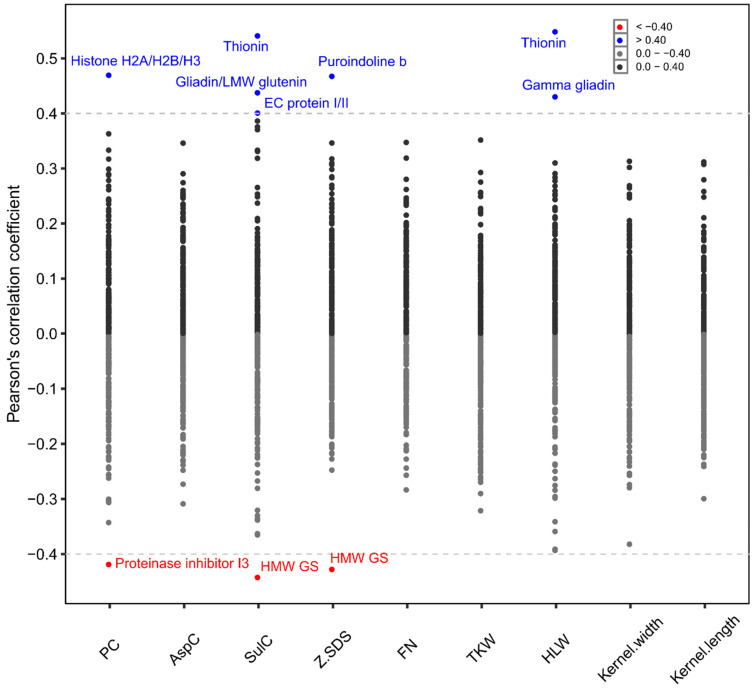
Correlation coefficients of the 299 proteins with quality traits (PC = total protein content, AspC = asparagine content, SulC = sulphur content, Z.SDS = Zeleny-SDS, FN = falling number, TKW = thousand kernel weight, and HLW = hectolitre weight). The dashed lines represent ± 0.40 correlation coefficients, and each point indicates a correlation coefficient between a single protein and a quality trait. LMW = low molecular weight, HMW = high molecular weight, and GS = glutenin subunit.

The variance components, heritability and Best Linear Unbiased Estimates (BLUEs) were estimated for 299 proteins, as the statistical model did not converge for four proteins. The highly significant variance components due to genotype and genotype-by-environment interactions were determined for 233 and 59 proteins, respectively (Table S1). BLUEs of different proteins varied largely (Figure 3a), but also, within each protein, a large variability of abundancy across the different cultivars was visible by the coefficients of variation (CV) ranging from 8.07% to 168.52% (Figure 3b). Similarly, a wide range of heritability was determined for the 299 proteins (Figure 3c). Here, 105 proteins had a heritability ≤0.40, 77 proteins between 0.41 and 0.60 and 117 proteins >0.60. The correlation coefficients between the proteins and typical quality traits of wheat were mostly weak (Figure 4).

As we investigated wheat cultivars registered between 1921 and 2013, we evaluated whether temporal trends in the expression of the proteins existed. According to the Kruskal-Wallis and Dunn’s tests, only 18 proteins revealed a temporal trend from old to modern wheat cultivars (Table 1). Eleven proteins showed an upward trend, seven proteins a downward trend and the other proteins no temporal trend and, thus, do not seem to have been affected by selection. Finally, we elaborated a list of proteins that (1) had a heritability >0.6, (2) had <20% missing data and (3) were expressed in >50% and >80% of the cultivars in three and two environments, respectively (Table S2). These proteins might be of interest for future research, as their expression levels could be influenced in a targeted manner along the wheat supply chain via varietal choice. Apart from many proteins with unknown functions, some proteins with functions potentially relevant for baking quality and human health were in this group and, therefore, are shown in Table 2.

## 3. Discussion

We used LC-MS-based LFQ proteomics to investigate extracts of the functionally relevant wheat albumins and globulins from 150 wheat cultivars registered between 1921 and 2013 that were grown in parallel in three diverse German environments.

### 3.1. Complex Interaction between Proteins, Cultivars and Environment

We detected, in total, 756 proteins across the 150 wheat cultivars grown in three environments (Figure 1a). However, only 303 proteins were stably expressed across all environments in at least one cultivar, while 453 proteins were expressed in only one or two environments, showing that their expression is mainly driven by environmental factors. We excluded these proteins from the further analyses and discussion. 

Within the 303 proteins that were stably expressed in at least one cultivar, we identified proteins with a different degree of expression stability across the cultivars and environments (Figure 1b and Figure 2a,b). For instance, there were 45 proteins that were stably expressed across the three environments in less than 25% of the wheat cultivars, while in the other cultivars, they were expressed only in two, one or even no environments (Figure 2b). Even more, the distribution of stable proteins in the cultivars differed for each protein, and only 89 proteins were stably expressed across the three environments and all cultivars without any missing data. 

These findings led to two main conclusions. First, the expression of proteins is largely affected by environmental factors. This is corroborated by several studies in the literature. Afzal et al. [36] showed that, from 3050 measured proteins, only 1604 were stably expressed in at least one of the 15 wheat cultivars. These authors also showed that heritability was quite low for many of the 1604 proteins. Another study reported that contents of gliadins, glutenins, albumins/globulins and peptides for celiac disease epitopes are affected more by the year of cultivation of the wheat cultivars than by the wheat cultivars themselves [26,29]. Similarly, classically determined kernel raw protein appears to be influenced largely by environmental factors such as nitrogen fertilization, weather conditions and soil types [2,4]. In line with these findings, the total protein content determined by NIRS technology in our dataset had a heritability of 0.65, while the sedimentation volume or the thousand kernel weight and hectoliter weight had heritability close to 0.9 [37]. 

Second, there exists a considerable variation in the expression of proteins across different cultivars, which is manifested either in a presence/absence of variation (Figure 1b) or, if present in many cultivars, in a large coefficient of variation across the cultivars (Figure 3b). These findings are in line with the results from Afzal et al. [36]. Consequently, protein profiles largely differ between cultivars, but the expression of that profile is also influenced by environmental factors, with varying importance of both factors. This underlines the large potential of proteomics for future wheat breeding and production but, also, the need for proteomic research to rely on an adequate number of different cultivars per species tested in several environments.

The BLUEs of the 299 proteins varied largely, with 43 proteins having a considerably higher abundancy than the majority of proteins (Figure 3a). Except three, the other highly abundant proteins were stable across the cultivars and environments, and most of them belong to four protein families, according to the InterPro database: Cupin1, Glycoside hydrolases, Bifunctional inhibitors/lipid transfer proteins/seed storage helical domain family and Cereal seed allergen/grain softness/bifunctional inhibitors/lipid transfer proteins/seed storage helical domain family. These protein families are large. According to InterPro, the proteins annotated as bifunctional inhibitors or cereal seed allergens belong to the “seed storage helical domain”. The Cupin superfamily consists of 18 different functional classes with functions including the modification of cell wall carbohydrates and plant growth and development [38]. Nevertheless, we found other members of these protein families that had lower abundancy. Furthermore, for the highly abundant proteins, considerable coefficients of variation across the cultivars and heritability fairly below 1 were detected, underlining the complex interaction of genotype and environment for highly abundant proteins. In particular, estimates of the heritability for the proteins of the Cereal seed allergen/grain softness protein family were medium-to-very low, indicating that their expression depends on particular microclimatic effects of the crop location and less on chosen cultivars, making their controlled manipulation across the wheat supply chain very difficult. According to the UniProt database, most of the highly abundant proteins in the Cereal seed allergen/grain softness protein family were ATIs, which stimulate, according to the literature, intestinal innate immune cells via the activation of Toll-like receptor 4 and promote intestinal and extraintestinal inflammation [12,13,14,15,16,17]. Thus, further research is warranted on applying the absolute quantification of important ATIs, for example, with isotope-labeled standard peptides [39].

### 3.2. Breeding for the Supply Chain

For the proteins that were environmental stably expressed in at least one cultivar, the mean values across the environments largely differed for the 150 cultivars, leading to large CVs (Figure 3b) and representing an important prerequisite for a successful breeding. Furthermore, heritability values ≥0.6 were determined for 124 of these proteins. Thus, for 16% of the initially determined number of proteins, breeding might be successful, as their expression can be considerably influenced by the varietal choice across wheat supply chains. Afzal et al. [36] determined that about 3% and 5% of detected proteins in spelt and bread wheat, respectively, had a heritability ≥0.6. Consequently, only a limited percentage of proteins, which are detectable with modern proteomic tools, appear to be influenced mainly by genetics, but these can be manipulated in a targeted way across the wheat supply chain and, thus, also be incorporated into future wheat breeding.

The correlation coefficients with common wheat quality traits were small for most of the 299 proteins. The highest coefficient of correlation was 0.54, which was determined for the association of prot290 (Q9S6Y2) with SulC, as well as with HLW (Figure 4). To our knowledge, however, a correlation coefficient of 0.54 shows only a moderate association. Consequently, none of the environmentally stable expressed proteins in our aqueous wheat extracts were associated with common wheat quality traits, enabling their incorporation into existing wheat breeding programs without drawbacks on already established traits. 

The varying degree of missing protein data across different cultivars and environments (Figure 1 and Figure 2) might lead to over/underestimation of their BLUEs, particularly for proteins with high amounts of missing data. For the interpretation and discussion of single proteins, we therefore applied a filter to keep only those proteins that had <20% missing data and that were expressed in >50% and >80% cultivars in three and two environments, respectively. This reduced the number of proteins for the final considerations further to 229. 

As our cultivar panel consisted of important wheat cultivars registered from 1921 to 2013, we investigated temporal trends for proteins due to selection over time. According to a Kruskal-Wallis and Dunn’s test, only eleven and seven proteins showed an upward and downward temporal trend, respectively (Table 1 and Figure S1). This is in contrast to the findings on important agronomic traits in wheat revealing that modern wheat cultivars have higher yields and better disease resistance than old wheat cultivars [40]. Similarly, it was demonstrated that modern wheat cultivars had higher sedimentation volumes but lower protein contents than old wheat cultivars [1]. The lack of a temporal trend for almost all proteins might be explained by the fact that they were not selected for either directly or indirectly by chance due to the absence of a close correlation with important selection traits like the protein content or sedimentation volume.

However, the detected upward trend for a few proteins towards a higher abundance in modern wheat cultivars seems logical. For instance, we found an upward trend for three proteins annotated as Gliadin/LMW glutenins in the InterPro database. This trend corroborates the findings of Pronin et al. [29], who reported an increase in the HMW and LMW glutenins across a set of 60 bread wheat cultivars registered during the last 100 years. Since the pioneering work of Payne et al. and Jackson et al. [27,28], wheat breeders around the world have intensively selected particular HMW/LMW subunits. 

We also found an upward trend from old to modern cultivars in the expression of heat shock protein (Hsp) 90 (Hsp90). Hsp90 has a multitude of proposed roles, including signal transduction, protein folding, protein degradation and growth and development under both normal and stressed conditions [41]. Previously, it was suggested that Hsp90 was implicated in controlling the seedling growth and resistance to stripe rust in wheat [42]. As modern wheat is selected for a high grain yield and high tolerance to biotic and abiotic stresses [40], the identified upward trend of Hsp90 might be due to breeder selection. Nevertheless, further work might be required to prove this deduction.

### 3.3. Application of New “Omics” Enables the Design of Better and Healthier Wheat 

Proteins with high heritability and high variability across different cultivars can successfully be manipulated across the wheat supply chain. We therefore extended our filter for missing data by the prerequisite for each protein to have a heritability >0.60, which gave us 114 proteins, presented in Table S2. Many of them have unfortunately no concrete functional annotation, several annotations are proposed for a single protein or their annotations differ between databases UniProt and InterPro, underpinning the necessity of research for the functional characterization of wheat proteins. However, for a few proteins, concrete annotations marking important functions were already available (Table 2). This involves a few proteins important for baking quality, e.g., three gliadins, two HMWs, two LMWs and puroindoline B. According to the database UniProt, the two HMWs were the alleles Dx5 (loci Glu-D1) and Dy9 (Glu-B1) known for their role in good baking quality [43]. Beside gluten proteins, puroindolines also influence the baking quality due to their effect on kernel hardness [44] and appear to correlate positively with the bread loaf volume [45], milling yield, dough extensibility and development time and water uptake of the flour [44].

Beside important proteins for baking quality, the list of 114 proteins that can be manipulated successfully across the supply chain also contained proteins known to be involved in human health, according to Juhász et al. [21] and Allergome—The Platform for Allergen Knowledge (http://www.allergome.org, accessed on 16 February 2021) (Table 2). This involves three β-amylases, one Bowman-Birk-type trypsin inhibitor, one ATI, two serpins, two nonspecific lipid transfer proteins and Hsp70 but, also, the three gliadins, two HMWs and LMWs and the puroindoline B mentioned above for the baking quality. Interestingly, prot139, an LMW, and prot245, a gliadin-like protein, showed an upward trend across the wheat cultivars registered between 1921 and 2013. This shows that demands for high baking quality might also negatively affect the health of sensitive humans, which is yet lowly regarded in research and completely disregarded in the wheat supply chain. Taking into account the high heritability and variability within wheat cultivars for these proteins, the establishment of proteomics in wheat research and breeding bears a large potential for designing wheat in the future with better quality and nutritional attributes suitable for sensitive humans warrants further research. 

In conclusion, high-throughput LC-MS methods for the label-free quantification of high numbers of proteins in wheat grains represent a very promising technology to deepen our knowledge on the important proteome characteristics in wheat. Many of the detected proteins are mainly influenced by the environment and, thus, likely cannot be manipulated in a targeted way in the wheat supply chain. Nevertheless, for proteins with high genetic variance, robust data and intermediate-to-high heritability, an efficient varietal choice along the supply chain and targeted breeding for them would be possible, as long as the market demands it. Such breeding efforts, however, would require optimized and cost-effective high-throughput proteomic workflows enabling proteomic characterizations within a few minutes to enable their implementation into the wheat supply chain. 

## 4. Materials and Methods

### 4.1. Plant Material and Field Trials

Details of the plant material, field trials and quality trait assessment were reported by Rapp et al. [37]. Briefly, 150 different European wheat cultivars (Table S3) were grown in three field locations Hohenheim (HOH, 48°43′07.3″ N 9°11′08.7″ E, 403 m above sea level (asl)—average annual temperature, precipitation and relative humidity were 10.12 °C, 595.4 mm and 81.67%, respectively); Eckartsweier (EWE, 48°32′52.4″ N 7°52′32.5″ E, 140 m asl—average annual temperature, precipitation and relative humidity were 10.96 °C, 538.9 mm and 87.01%, respectively) and Oberer Lindenhof (OLI, 48°28′19.0″ N 9°18′29.3″ E, 700 m asl—average annual temperature, precipitation and relative humidity were 8.5 °C, 779.3 mm and 85.93%, respectively) during wheat cultivation season 2015/2016 in a partially replicated (p-rep) design. The type of soil of locations HOH, EWE and OLI is loamy sand, brown earth and clayey loam, respectively. The following quality traits were assessed: Asparagine content was measured in mg/kg (AspC, European Commission Regulation—EC No 152/2009, Annex III, Method F; for details, see Rapp et al. [37]), sulphur content in mg/kg (SulC, Elementar Analysensysteme GmbH, Langenselbold, Germany), total protein content in % (PC, ICC standard method 159, ICC, Vienna, Austria), sedimentation value in mL according to Zeleny (Z-SDS, ICC standard method 116/1, ICC, Vienna, Austria), falling number measured in seconds (FN, ICC standard method 107/1, Vienna, Austria) and thousand kernel weight in grams (TKW), as well as kernel width and length measured in mm using a Marvin seed analyzer (GTA Sensorik, Neubrandenburg, Germany). Hectolitre weight was measured in kg (HLW) by weight of grains fitting in a cylindrical can with a volume of 26 mL, as HLW = (grain weight (kg)/volume (L)) * 100 L. 

### 4.2. Quantitative Proteomic Analyses

LFQ proteomic analyses using LC-MS were performed as described previously by Sielaff et al. [39]. In this study, our aim was to set up a high-throughput method, allowing single-shot LC-MS analyses by keeping the run-to-run variation as small as possible by using a robust and fast microflow LC setup (compared to the more fragile nanoflow LC commonly used in proteomics) and a non-stochastic, data-independent acquisition method [39]. Fine whole grain flour of the harvested samples served as the starting material, which was generated using the Cyclotec Laboratory Mill (Foss GmbH, Hamburg, Germany) and stored at −20 °C until extraction. Extraction protocols were chosen with the primary goal to investigate the reactions in cell cultures for wheat sensitivity. Native water/salt-soluble proteins, i.e., albumins and globulins (and minor amounts of gliadins and glutenins), represent up to 20% of the wheat proteome [46], and, thus, we also used these extracts for proteomic profiling of the wheat cultivars. In particular, these proteins were quantitatively extracted from 1 g of wheat flour using 5 mL of 10-mM sodium bicarbonate and 500-mM sodium chloride, pH 7.8, with constant spinning at 4 °C overnight. After centrifugation at 4600× *g* for 30 min, the supernatant was collected, and the extraction was repeated from the pre-extracted pellet. Both supernatants were combined and sterile-filtered (0.22 µm).

Sample preparation for the proteome analysis was performed in a 96-well format. Five microliters (µL) of protein extracts (~5 µg of total protein) were diluted in 50-mM ammonium bicarbonate and 0.1% (*w/v*) RapiGest surfactant (Waters Corporation, Milford, MA, USA) to a final volume of 50 µL and incubated at 80 °C for 15 min. Disulphide bridges were reduced by adding dithiothreitol (DTT) to a final concentration of 8 mM and incubating the samples at 56 °C for 1 h. Afterwards, cysteines were alkylated by adding iodoacetamide (IAA) to a final concentration of 16 mM and incubation in the dark at 22 °C for 30 min. Excess IAA was quenched by adding DTT to a final concentration of 8 mM. Proteins were digested using trypsin (Trypsin Gold, Mass Spectrometry Grade, Promega, Madison, WI, USA) at a protease-to-protein ratio of 1:25 (*w/w*) and incubated overnight at 37 °C. The reaction was stopped by acidifying the samples using 0.5% (*v/v*) trifluoroacetic acid (TFA). After incubation at 37 °C for 45 min, hydrolysed RapiGest was pelleted by centrifugation at 4600× *g* at 22 °C for 30 min. Supernatants were desalted using Sep-Pak tC_18_ cartridges (Waters Corporation), and purified peptides were eluted using 50% (*v/v*) acetonitrile (ACN) and 0.1% (*v/v*) TFA in water. Samples were lyophilized and reconstituted in 20 µL of 0.1% (*v/v*) formic acid (FA) prior to liquid chromatography-mass spectrometry (LC-MS) analyses.

LC-MS measurements were carried out using a nanoACQUITY Ultra-Performance Liquid Chromatography (UPLC) system (Waters Corporation) online coupled to a SYNAPT G2-S mass spectrometer (Waters Corporation) via a NanoLockSpray dual electrospray ionization (ESI) source (Waters Corporation). A sample amount of 0.5 µL was loaded onto a HSS T3 300 µm × 100 mm, 1.8-µm reversed-phase column (Waters Corporation). Peptides were separated at a flow rate of 8 µL/min over 15 min using a gradient of 1 to 36% (*v/v*) solvent B. Water with 0.1% (*v/v*) FA was used as solvent A, while ACN with 0.1% (*v/v*) FA was used as solvent B. The column temperature was kept at 55 °C. To enhance the ESI process, 25% (*v/v*) dimethyl sulfoxide (DMSO) at a flow rate of 1 µL/min was added post-column to the eluting peptides, as detailed before [47]. The lock mass compound [Glu1]-Fibrinopeptide B was directly delivered to the reference sprayer of the ESI source at a concentration of 250 fmol/µL and a flow rate of 1.5 µL/min.

Acquisition of mass spectra was performed in data-independent mode (DIA) by alternating between low (MS) and high-energy scans (MS^E^) [48]. The quadrupole was set to transmit all ions of *m*/*z* 50–1990. Scan time was 0.4 s, with a 0.05-s interscan delay for MS, as well as MS^E^, scans, resulting in an overall cycle time of 0.9 s. MS scans were acquired at a collision energy of 4 eV, while the collision energy was ramped from 16 to 40 eV during the MS^E^ scans. The reference sprayer was sampled every 30 s, and the doubly charged monoisotopic ion of [Glu1]-Fibrinopeptide B was used for post-acquisition lock mass correction.

Each sample was analyzed once by LC-MS, resulting in 486 sample measurements. In order to monitor the system stability and reproducibility of the LC-MS measurements, a tryptic digest of HeLa cell lysate was repeatedly injected between the sample runs and analyzed using the same LC-MS method as described before.

Raw data were processed using ProteinLynx Global Server v2.0.3 (PLGS, Waters Corporation) and searched against the UniProt *T. aestivum* protein database (UniProtKB release 2019_02, taxon ID: 4565, 142,700 entries) concatenated with *Escherichia coli* proteins (UniProtKB/Swiss-Prot release 2019_02, taxon ID: 83333, 4550 entries) and 171 common MS contaminants. Trypsin was set as the digestion enzyme, with a maximum of two missed cleavages. The carbamidomethylation of cysteine was set as the fixed modification, while the oxidation of methionine was set as the variable modification. The false discovery rate (FDR) was estimated by searching a reversed-sequence database.

Postprocessing and label-free quantification (LFQ), including retention time alignment, feature clustering, multidimensional local regression-based normalization and protein homology filtering, were performed using ISOQuant v1.8 [49]. Samples of each location (HOH, EWE and OLI) were processed separately in ISOQuant, dividing the dataset into three subsets. Peptides with a minimum ProteinLynx Global Server database search score of 6.0, a minimum sequence length of six amino acids, no missed cleavages and no methionine oxidations were considered for LFQ. Proteins were only reported if they were identified by at least two peptides. An FDR cut-off of 0.01 was used at the peptide and protein level. Protein quantities were calculated as the mean signal of the three most-intense peptides matching each protein (Top3) [50].

The 13 repeated measurements of the HeLa standard were processed in PLGS (searched against a *Homo sapiens* UniProtKB/Swiss-Prot protein database) and ISOQuant in the same way as described for the samples in order to confirm the high stability of the LC-MS system and reproducibility of the measurements during the course of the experiment (Pearson correlation coefficient of LFQ protein abundances >0.99 for all run-to-run comparisons and median coefficient of variation <5%).The mass spectrometry proteomics data were deposited to the ProteomeXchange Consortium (http://proteomecentral.proteomexchange.org, accessed on 18 January 2021) via the PRIDE [51,52] partner repository with the dataset identifier PXD023654.

### 4.3. Phenotypic Data Analysis

Phenotypic data analysis was performed according to the statistical model given in Equation (1):

(1) $$y_{ikno}=u+g_{i}+env_{k}+g_{i}:env_{k}+rep_{kn}+b_{kno}+e_{ikno}\;,$$

where $y_{ikno}$ was the phenotypic observation for the *i*th genotype tested in the *k*th environment in the *n*th replication in the *o*th incomplete block, u was the general mean, $g_{i}$ the genotypic effect of the *i*th genotype, $env_{k}$ the effect of the *k*th environment, $g_{i}:env_{k}$ was the genotype-by-environment interaction, $rep_{kn}$ was the effect of the *n*th replication in the *k*th environment, $b_{kno}$ was the effect of the *o*th incomplete block of the *n*th replication in the *k*th environment and $e_{ikno}$ was the residual.

Variance components were estimated using the restricted maximum likelihood (REML) method assuming a random model in a classical one-stage analysis [53]. A likelihood ratio test with model comparisons was performed [54] to check for significance of the variance components. Best Linear Unbiased Estimates (BLUEs) were estimated across the environments assuming fixed genotypic effects. Broad sense heritability (*h*^2^) was estimated as given in Equation (2):

(2) $$h^{2}=1-\frac{\vartheta }{2\sigma_{G}^{2}}\;,$$

where ϑ is the mean variance of a difference of the two best linear unbiased predictors and $\sigma_{G}^{2}$ the genotypic variance [55]. Pearson’s correlation coefficients ($r_{p}$) were estimated among the BLUEs of the examined traits. All analyses were performed utilizing the statistical software R [56] and the software ASReml 3.0 [57].

### Detection of Temporal Trend

To test whether the expression of proteins changed due to breeding, we compared cultivars from different registration periods using the nonparametric Kruskal-Wallis test [58] and Dunn’s test [59]. For multiple comparisons, the *p*-value was adjusted using the Holm’s method. Kruskal-Wallis test and Dunn’s test were implemented using the R-package *rstatix*. We chose nonparametric tests over the standard analysis of variance (ANOVA), because the sample sizes between the six groups of varietal registration periods varied greatly from 13 (1991–2000) to 53 (2001–2013). Other groups such as 1921–1960, 1961–1970, 1971–1980 and 1981–1990 had 22, 16, 22 and 23 samples, respectively. As a result, the assumptions of ANOVA, i.e., data approximately normally distributed, sufficiently large sample size (usually *n* > 30) and homoscedastic variance in the comparison groups, could not be applied.

## Figures and Tables

**Table 1:** List of proteins stably expressed across all environments in at least one cultivar that showed a temporal trend across wheat cultivars that were registered during the last 100 years.

IPN.	UniProt Accession	UniProt Annotation	InterPro Annotation	Heritability	Missing Data ^a^ (%)	PV2 ^b^ (%)	PV3 ^c^ (%)
prot034 ^u^	A0A3B6EGL9	Uncharacterized	Aspartic peptidase A1 family;Saposin-like type B	0.65	0.21	100	99
prot036 ^d^	A0A3B6MYZ0	Uncharacterized	Cupin 1;RmlC-like jelly roll fold	0.72	0.21	100	100
prot045 ^u^	A0A3B6JER7	Uncharacterized	Cupin 1;RmlC-like jelly roll fold	0.83	0	100	100
prot123 ^u^	A0A3B6HSA4	Uncharacterized	Cupin 1;RmlC-like jelly roll fold	0.68	0	100	100
prot156 ^d^	A0A3B6LUV8	Uncharacterized	Cupin 1;RmlC-like jelly roll fold	0.71	0	100	100
prot139 ^u^	I1XB56	Low-molecular-weight glutenin subunit	Bifunctional inhibitor/plant lipid transfer protein/seed storage helical domain superfamily… Gliadin/LMW glutenin	0.83	8.85	90	84
prot245 ^u^	D2KFG9	Gliadin/avenin-like seed protein	Bifunctional inhibitor/plant lipid transfer protein/seed storage helical domain superfamily… Gliadin/LMW glutenin	0.61	0	100	100
prot292 ^u^	R9YQY9	Low-molecular-weight glutenin subunit Glu-D3	Bifunctional inhibitor/plant lipid transfer protein/seed storage helical domain superfamily… Gliadin/LMW glutenin	0.45	0.62	100	98
prot069 ^u^	A0A3B6C1C0	Uncharacterized	Glycoside hydrolase family 1	0.75	0	100	100
prot073 ^u^	A0A1D5V0T8	Uncharacterized	Glycoside hydrolase family 1	0.69	0.21	100	99
prot018 ^u^	F4Y589	Heat shock protein 90	Heat shock protein Hsp90	0.60	0.21	100	99
prot218 ^d^	P02276	Histone H2A.2.1	Histone-fold;Histone H2A	0.67	0	100	100
prot240 ^d^	Q9SWU3	Histone H1 WH1A.1	Histone H1/H5	0.55	0	100	100
prot222 ^u^	A0A024CKY0	LEA protein	Late embryogenesis abundant protein, LEA_4 subgroup	0.34	2.06	100	93
prot093 ^d^	P30570	EC protein III	Plant EC metallothionein-like protein, family 15	0.64	4.94	100	84
prot068 ^u^	A0A3B5YRZ8	Uncharacterized	Proteinase inhibitor I13	0.58	12.76	91	74
prot102 ^d^	A0A3B6JQP1	Uncharacterized	Cereal seed allergen/grain softness/Trypsin and alpha-amylase inhibitor	0.25	0	100	100
prot233 ^d^	A0A3B6SMC2	Uncharacterized	Ubiquitin domain;Ubiquitin-like domain superfamily	0.13	0.21	100	99

IPN = internal protein number. ^u^ Protein showed upward temporal trend, ^d^ protein showed downward temporal trend, ^a^ the proportion of missing raw data (i.e., protein not detected in a sample), ^b^ the proportion of 150 cultivars in which a protein was detected in at least two of the three environments, and ^c^ the proportion of 150 cultivars in which a protein was detected in all three environments.

**Table 2:** List of proteins stably expressed across all environments in at least one cultivar and that had a heritability >0.6, had <20% missing data and were expressed in > 50% and > 80% of the cultivars in three and two environments, respectively, and where a potentially relevant function for baking quality and human health is assigned in the UniProt or InterPro databases. Although a few proteins have similar names, they were each independently quantified by several unique peptides.

IPN	UniProt Accession	UniProt Annotation	InterPro Annotation	Heritability	CV (%)	Missing Data ^a^ (%)	PV2 ^b^ (%)	PV3 ^c^ (%)
prot085	Q7X9M2	Beta-amylase (Fragment)	Glycoside hydrolase, family 14	0.74	31.77	1.65	100	95
prot141	A0A3B6KSH4	Beta-amylase	Glycoside hydrolase, family 14	0.85	36.89	0	100	100
prot144	A0A3B6IYD4	Beta-amylase	Glycoside hydrolase, family 14	0.81	25.07	0	100	100
prot028	Q94G97 ^ⴕ^	Gamma-gliadin	Bifunctional inhibitor/plant lipid transfer protein/seed storage helical domain superfamily… Gliadin/LMW glutenin	0.91	59.97	15.64	81	72
prot066	D2KFH0 ^ⴕ^	Gliadin/avenin-like seed protein	Bifunctional inhibitor/plant lipid transfer protein/seed storage helical domain superfamily… Gliadin/LMW glutenin	0.83	59.31	19.96	81	61
prot245	D2KFG9 ^ⴕ^	Gliadin/avenin-like seed protein	Bifunctional inhibitor/plant lipid transfer protein/seed storage helical domain superfamily… Gliadin/LMW glutenin	0.61	23.30	0	100	100
prot017	G1E6K7 ^ⴕ^	High molecular weight glutenin subunit Dx5 (Fragment)	Bifunctional inhibitor/plant lipid transfer protein/seed storage…HMW glutenin	0.76	41.79	1.65	100	95
prot103	Q03871 ^ⴕⴕ^	HMW glutenin subunit 1By9	Cereal seed allergen/grain softness/trypsin and alpha-amylase inhibitor… HMW glutenin	0.65	40.63	6.38	96	85
prot139	I1XB56 ^ⴕ^	Low-molecular-weight glutenin subunit (Fragment)	Bifunctional inhibitor/plant lipid transfer protein/seed storage …LMW-GS	0.83	46.97	8.85	90	84
prot203	C3VN75 ^ⴕ^	Low molecular weight glutenin GN = Glu-A3	Bifunctional inhibitor/plant lipid transfer protein/seed storage …LMW-GS	0.67	55.83	15.23	89	65
prot044	A0A3B6HWI6	Uncharacterized	Heat shock protein 70, conserved site	0.66	22.21	7.20	96	83
prot009	H2DLU3 ^ⴕ^	Puroindoline b GN = Pinb-D1	Cereal seed allergen/grain softness/trypsin and alpha-amylase inhibitor	0.92	67.59	3.29	97	93
prot110	P81713	Bowman-Birk type trypsin inhibitor	Bowman-Birk type proteinase inhibitor	0.90	76.72	15.23	83	68
prot030	Q43691 ^ⴕ^	Trypsin/alpha-amylase inhibitor CMX2	Cereal seed allergen/grain softness/trypsin and alpha-amylase inhibitor	0.64	25.12	12.96	97	61
prot072	Q0Q5D9 ^ⴕ^	Globulin 1	Cereal seed allergen/grain softness/trypsin and alpha-amylase inhibitor	0.66	18.68	0	100	100
prot288	A0A1D5UB33 ^ⴕ^	Uncharacterized	Cereal seed allergen/grain softness/trypsin and alpha-amylase inhibitor	0.95	106.86	0	100	100
prot189	Q2PCC3 ^ⴕⴕ^	Type 2 non specific lipid transfer protein	Bifunctional inhibitor/plant lipid transfer protein/seed storage helical domain superfamily	0.85	32.02	3.70	98	90
prot235	Q2PCC5 ^ⴕⴕ^	Type 2 non specific lipid transfer protein	Bifunctional inhibitor/plant lipid transfer protein/seed storage helical domain superfamily	0.93	28.71	0	100	100
prot232	A0A3B6TLW2	Uncharacterized	Serpin, conserved site;Serpin domain	0.81	24.60	0.62	100	98
prot287	A0A3B6LS85	Uncharacterized	Serpin, conserved site;Serpin domain	0.66	27.39	9.88	98	70

IPN = internal protein number and CV = coefficient of variation. ^a^ The proportion of missing raw data (i.e., protein not detected in a sample), ^b^ the proportion of 150 cultivars in which a protein was detected in at least two of the three environments, ^c^ the proportion of 150 cultivars in which a protein was detected in all three environments, ^ⴕ^ protein listed as an allergen by Juhász et al. [21] and ^ⴕⴕ^ protein listed as an allergen according to the Allergome database (http://www.allergome.org, accessed on 16 February 2021).

## Data Availability

The mass spectrometry proteomics data are available via ProteomeXchange with identifier PXD023654.

## Supplementary Materials

The following are available online at https://www.mdpi.com/2223-7747/10/3/424/s1: Figure S1: Boxplots of proteins that showed temporal trends. Table S1: Summary of the phenotypic analysis of the proteins. Table S2: List of proteins for future breeding and research. Table S3: List of the 150 wheat cultivars used in this study.
