# Supplementary material for: Characterization of 150 Wheat Cultivars by LC-MS-Based Label-Free Quantitative Proteomics Unravels Possibilities to Design Wheat Better for Baking Quality and Human Health
Source: Plants (Basel). 2021 Feb 24;10(3):424. doi: 10.3390/plants10030424 (PMC7996164; doi:10.3390/plants10030424)
Supplement: Supplementary file 1 [file plants-10-00424-s001.zip › Figure S1_Boxplots of proteins that showed temporal trend.pdf]

Characterization of 150 wheat cultivars by LC-MS based label free quantitative proteomics unravels possibilities to design wheat better for baking quality and human health

Muhammad Afzal[1], Malte Sielaff[2], Valentina Curella[3], Manjusha Neerukonda[3], Khaoula El Hassouni[1], Detlef Schuppan[3,4], Stefan Tenzer[2] and C. Friedrich H. Longin*[1]

[1]State Plant Breeding Institute, University of Hohenheim, Fruwirthstr. 21, 70599 Stuttgart, Germany

[2]Institute for Immunology, University Medical Center of the Johannes Gutenberg University Mainz, Langenbeckstr. 1, 55131 Mainz, Germany

[3]Institute of Translational Immunology, University Medical Center of the Johannes Gutenberg University Mainz, Langenbeckstr. 1, 55131 Mainz, Germany

[4]Division of Gastroenterology, Beth Israel Deaconess Medical Center, Harvard Medical School, 330 Brookline Avenue, Boston, MA 02215, USA

*Corresponding author: C. Friedrich H. Longin, E-mail: friedrich.longin@uni-hohenheim.de

Supplementary Figure S1: Boxplots of proteins that showed temporal trend

According to Dunn's test, *, **, and *** indicate significant pairwise differences at adjusted *p* value of < 0.05, < 0.01 and < 0.001, respectively. Lines for non-significant pairwise differences are not shown. Group means are denoted by red triangles. For each protein, the UniProt accession is appended to the internal protein number after "_".

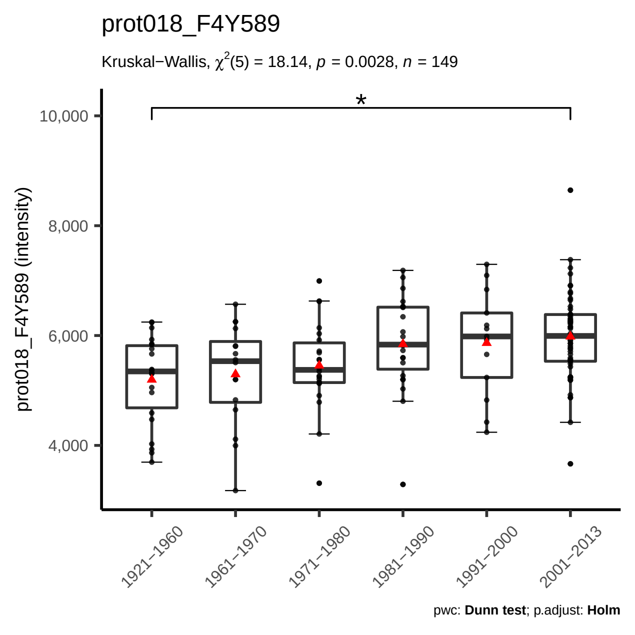

prot018_F4Y589
Kruskal−Wallis, $\chi^2(5) = 18.14$, $p = 0.0028$, $n = 149$
*
prot018_F4Y589 (intensity)
10,000
8,000
6,000
4,000
1921−1960
1961−1970
1971−1980
1981−1990
1991−2000
2001−2013
pwc: **Dunn test**; p.adjust: **Holm**

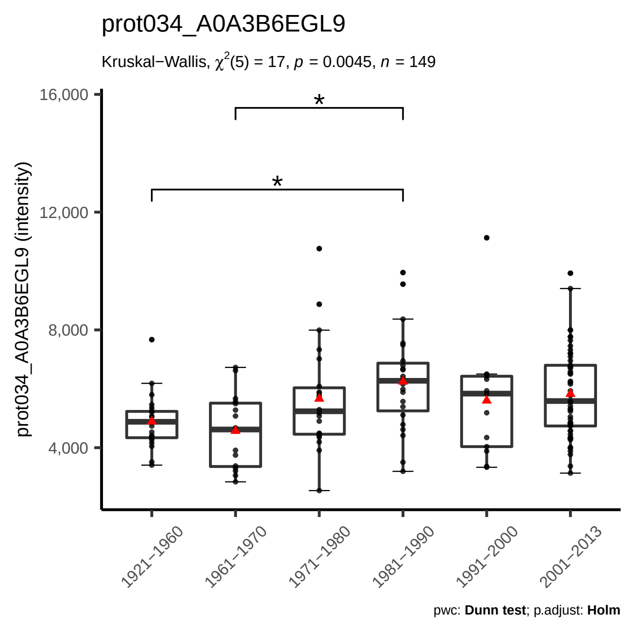

prot034_A0A3B6EGL9
Kruskal−Wallis, χ2(5) = 17, p = 0.0045, n = 149
prot034_A0A3B6EGL9 (intensity)
16,000
12,000
8,000
4,000
*
*
1921−1960
1961−1970
1971−1980
1981−1990
1991−2000
2001−2013
pwc: Dunn test; p.adjust: Holm

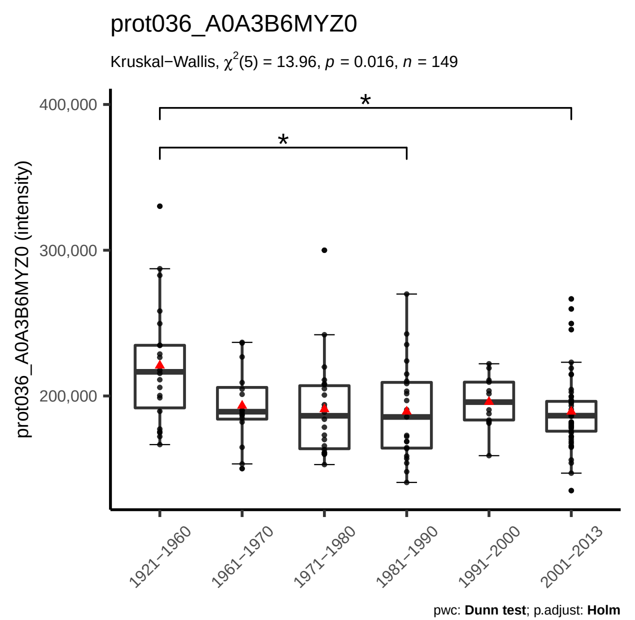
prot036_A0A3B6MYZ0
Kruskal−Wallis, $\chi^2(5)$ = 13.96, p = 0.016, n = 149
400,000
300,000
200,000
prot036_A0A3B6MYZ0 (intensity)
*
*
1921−1960
1961−1970
1971−1980
1981−1990
1991−2000
2001−2013
pwc: **Dunn test**; p.adjust: **Holm**

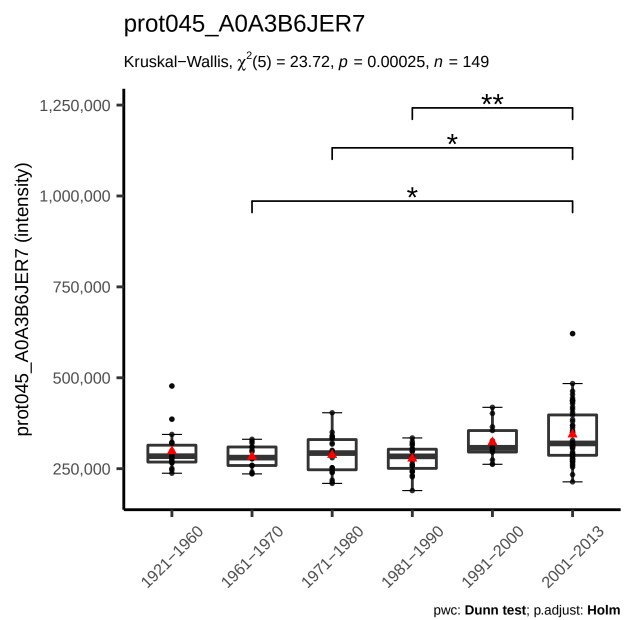

prot045_A0A3B6JER7
Kruskal−Wallis, χ2(5) = 23.72, p = 0.00025, n = 149
**
*
*
1,250,000
1,000,000
750,000
500,000
250,000
prot045_A0A3B6JER7 (intensity)
1921−1960
1961−1970
1971−1980
1981−1990
1991−2000
2001−2013
pwc: Dunn test; p.adjust: Holm

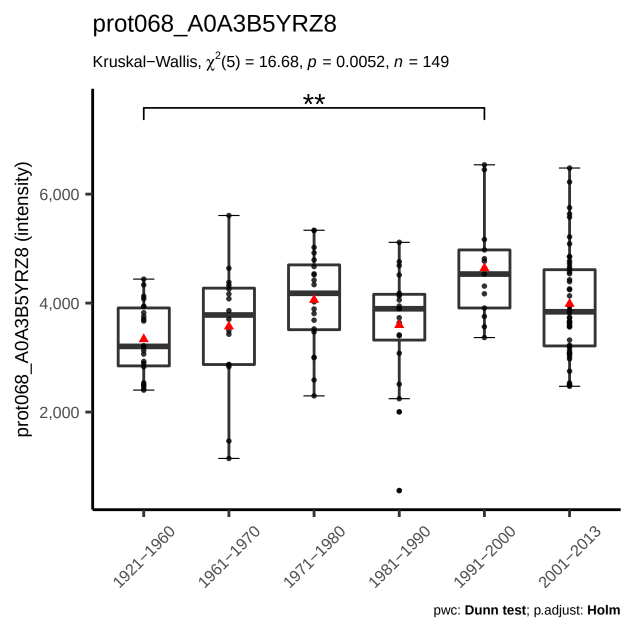
prot068_A0A3B5YRZ8
Kruskal−Wallis, χ2(5) = 16.68, p = 0.0052, n = 149
**
prot068_A0A3B5YRZ8 (intensity)
6,000
4,000
2,000
1921−1960
1961−1970
1971−1980
1981−1990
1991−2000
2001−2013
pwc: Dunn test; p.adjust: Holm

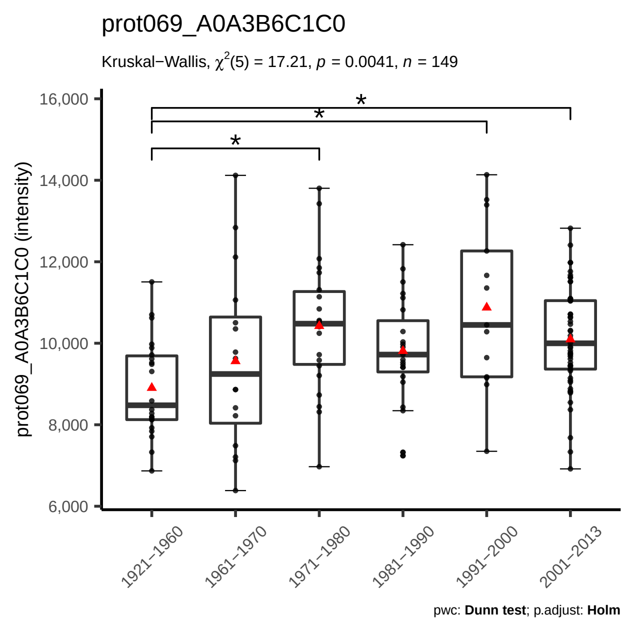
prot069_A0A3B6C1C0
Kruskal−Wallis, χ2(5) = 17.21, p = 0.0041, n = 149
16,000
14,000
12,000
10,000
8,000
6,000
prot069_A0A3B6C1C0 (intensity)
*
*
*
1921−1960
1961−1970
1971−1980
1981−1990
1991−2000
2001−2013
pwc: Dunn test; p.adjust: Holm

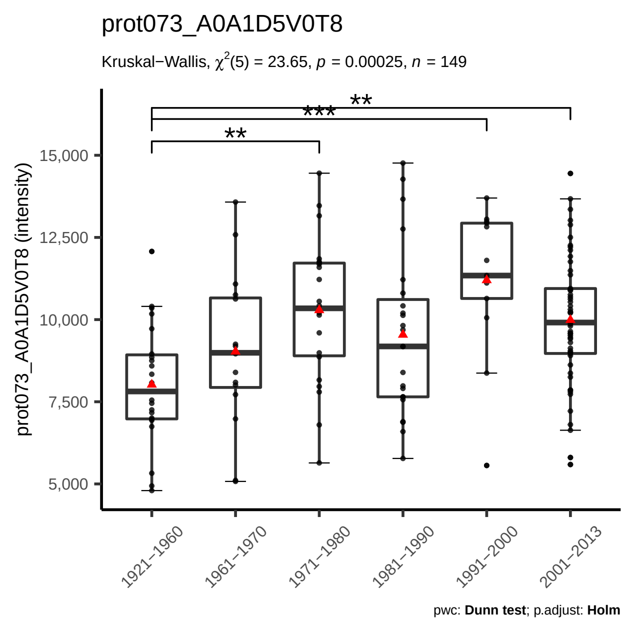

prot073_A0A1D5V0T8
Kruskal−Wallis, $\chi^2(5) = 23.65$, $p = 0.00025$, $n = 149$
**
***
**
15,000
12,500
10,000
7,500
5,000
prot073_A0A1D5V0T8 (intensity)
1921−1960
1961−1970
1971−1980
1981−1990
1991−2000
2001−2013
pwc: **Dunn test**; p.adjust: **Holm**

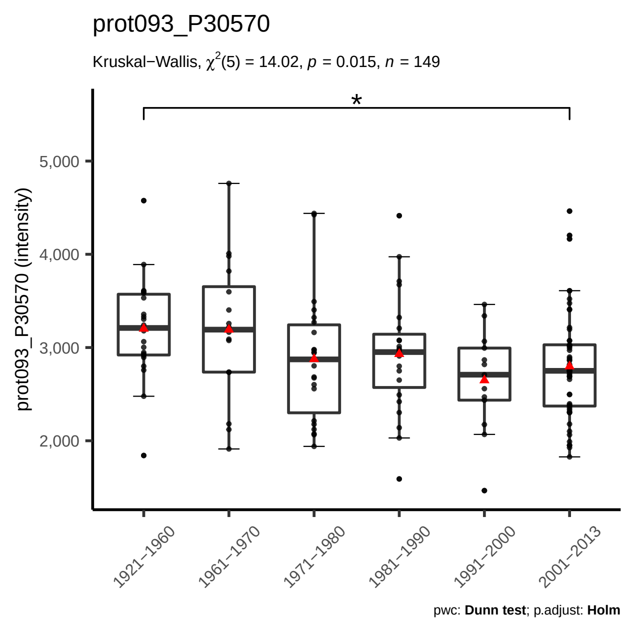

prot093_P30570
Kruskal−Wallis, $\chi^2(5) = 14.02$, $p = 0.015$, $n = 149$
*
5,000
4,000
3,000
2,000
prot093_P30570 (intensity)
1921−1960
1961−1970
1971−1980
1981−1990
1991−2000
2001−2013
pwc: **Dunn test**; p.adjust: **Holm**

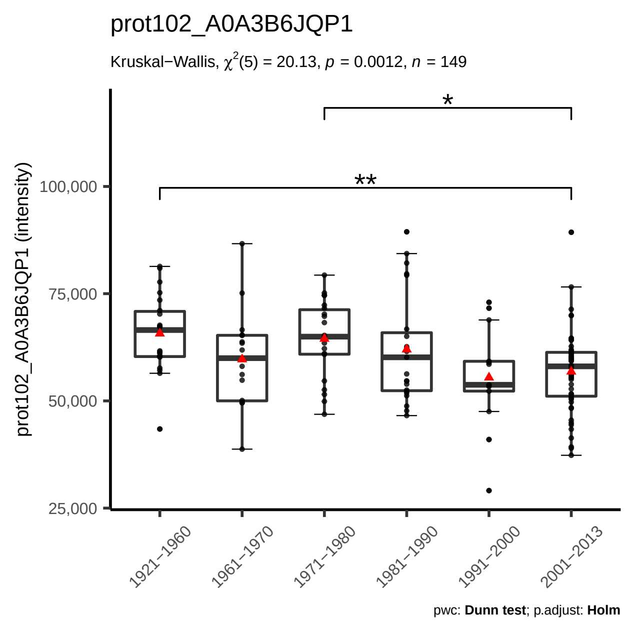
prot102_A0A3B6JQP1
Kruskal−Wallis, $\chi^2(5)$ = 20.13, p = 0.0012, n = 149
*
**
100,000
75,000
50,000
25,000
prot102_A0A3B6JQP1 (intensity)
1921−1960
1961−1970
1971−1980
1981−1990
1991−2000
2001−2013
pwc: **Dunn test**; p.adjust: **Holm**

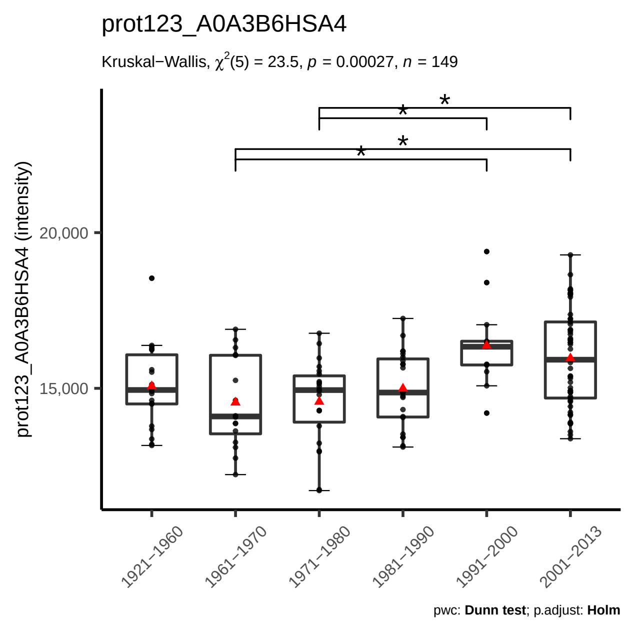
prot123_A0A3B6HSA4
Kruskal−Wallis, $\chi^2(5) = 23.5$, $p = 0.00027$, $n = 149$
prot123_A0A3B6HSA4 (intensity)
20,000
15,000
*
*
*
*
1921−1960
1961−1970
1971−1980
1981−1990
1991−2000
2001−2013
pwc: **Dunn test**; p.adjust: **Holm**

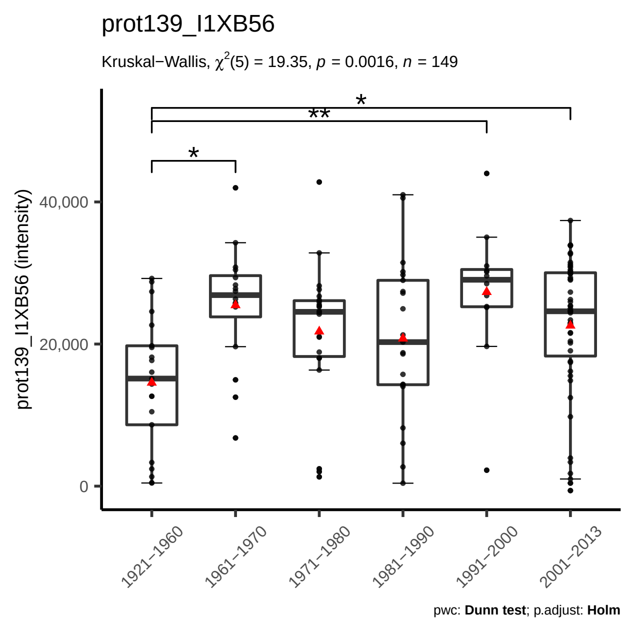

prot139_I1XB56
Kruskal−Wallis, $\chi^2(5) = 19.35$, $p = 0.0016$, $n = 149$
prot139_I1XB56 (intensity)
40,000
20,000
0
*
**
*
1921–1960
1961–1970
1971–1980
1981–1990
1991–2000
2001–2013
pwc: **Dunn test**; p.adjust: **Holm**

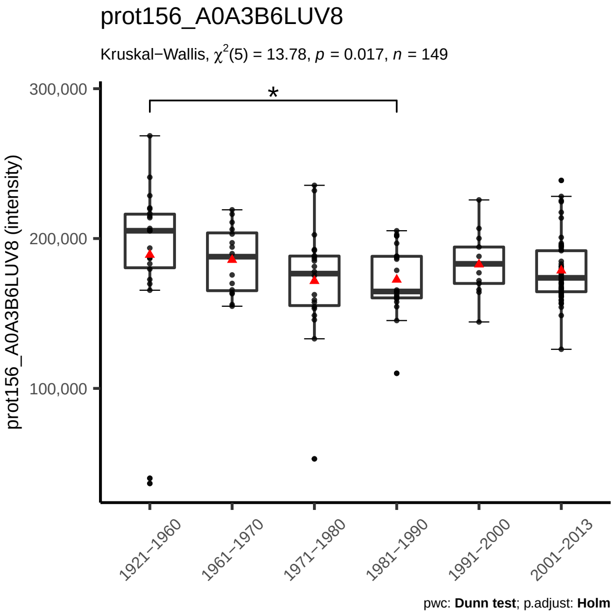

prot156_A0A3B6LUV8
Kruskal−Wallis, $\chi^2(5)$ = 13.78, p = 0.017, n = 149
*
300,000
200,000
100,000
prot156_A0A3B6LUV8 (intensity)
1921−1960
1961−1970
1971−1980
1981−1990
1991−2000
2001−2013
pwc: **Dunn test**; p.adjust: **Holm**

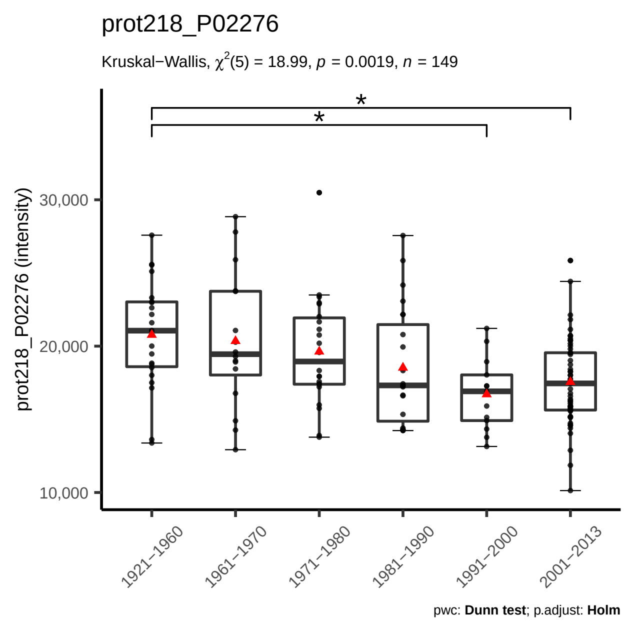

prot218_P02276
Kruskal−Wallis, $\chi^2(5)$ = 18.99, p = 0.0019, n = 149
*
*
30,000
20,000
10,000
prot218_P02276 (intensity)
1921−1960
1961−1970
1971−1980
1981−1990
1991−2000
2001−2013
pwc: **Dunn test**; p.adjust: **Holm**

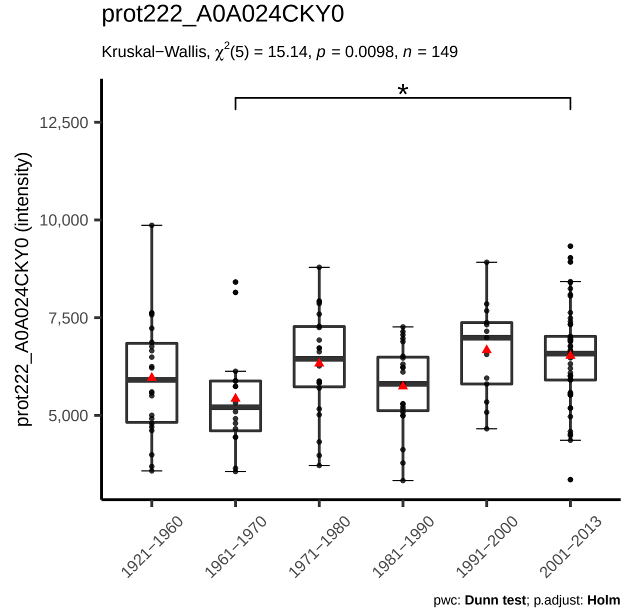
prot222_A0A024CKY0
Kruskal−Wallis, χ2(5) = 15.14, p = 0.0098, n = 149
*
12,500
10,000
7,500
5,000
prot222_A0A024CKY0 (intensity)
1921−1960
1961−1970
1971−1980
1981−1990
1991−2000
2001−2013
pwc: Dunn test; p.adjust: Holm

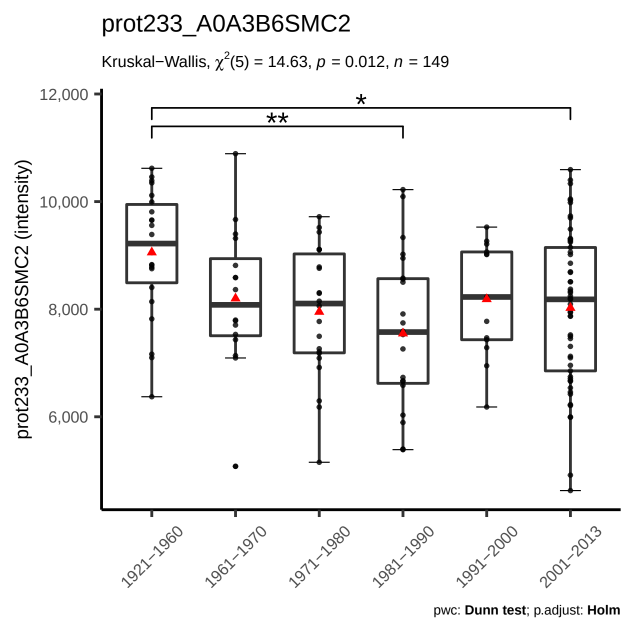

prot233_A0A3B6SMC2
Kruskal−Wallis, $\chi^2(5) = 14.63$, $p = 0.012$, $n = 149$
12,000
10,000
8,000
6,000
prot233_A0A3B6SMC2 (intensity)
*
**
1921–1960
1961–1970
1971–1980
1981–1990
1991–2000
2001–2013
pwc: **Dunn test**; p.adjust: **Holm**

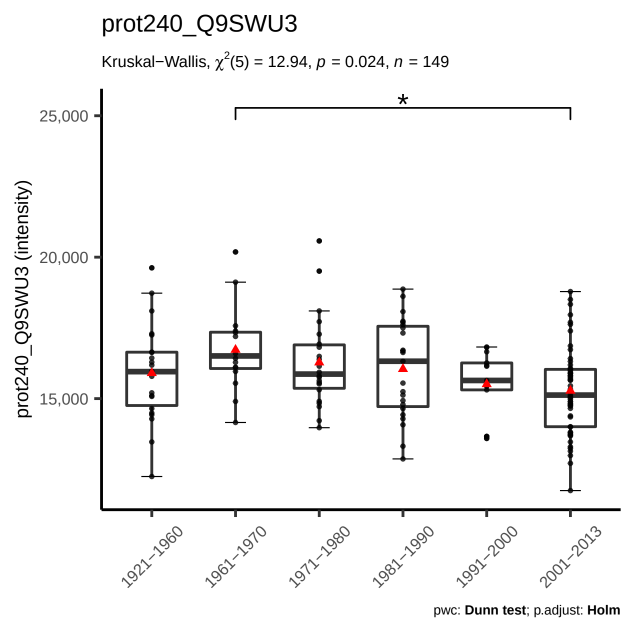
prot240_Q9SWU3
Kruskal−Wallis, $\chi^2(5)$ = 12.94, p = 0.024, n = 149
*
prot240_Q9SWU3 (intensity)
25,000
20,000
15,000
1921−1960
1961−1970
1971−1980
1981−1990
1991−2000
2001−2013
pwc: **Dunn test**; p.adjust: **Holm**

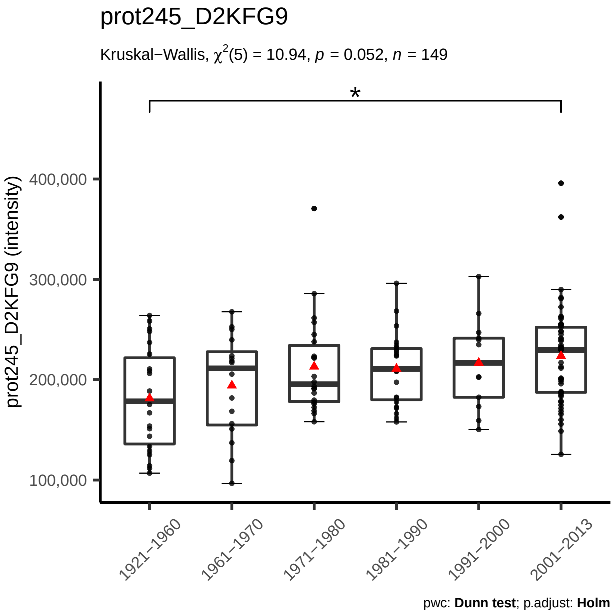
prot245_D2KFG9
Kruskal−Wallis, $\chi^2(5)$ = 10.94, p = 0.052, n = 149
*
prot245_D2KFG9 (intensity)
400,000
300,000
200,000
100,000
1921−1960
1961−1970
1971−1980
1981−1990
1991−2000
2001−2013
pwc: **Dunn test**; p.adjust: **Holm**

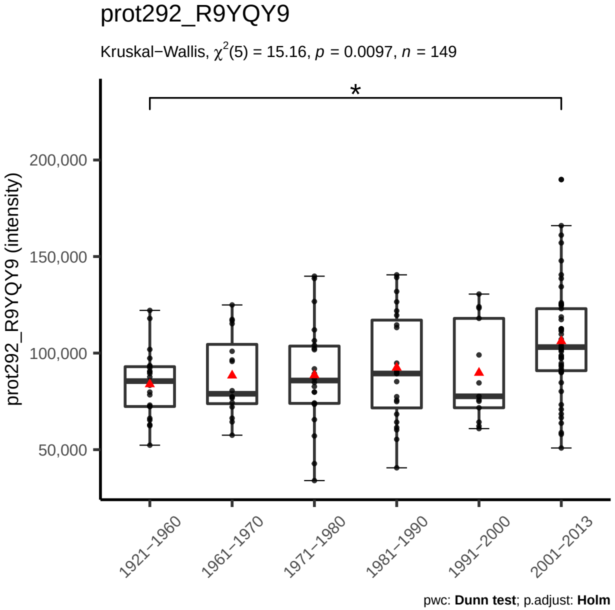
prot292_R9YQY9
Kruskal−Wallis, $\chi^2(5) = 15.16$, $p = 0.0097$, $n = 149$
*
200,000
150,000
100,000
50,000
prot292_R9YQY9 (intensity)
1921−1960
1961−1970
1971−1980
1981−1990
1991−2000
2001−2013
pwc: **Dunn test**; p.adjust: **Holm**
